# Multifactorial Origin of Exertional Rhabdomyolysis, Recurrent Hematuria, and Episodic Pain in a Service Member with Sickle Cell Trait

**DOI:** 10.1155/2018/6898546

**Published:** 2018-11-07

**Authors:** Nyamkhishig Sambuughin, Mingqiang Ren, John F. Capacchione, Ognoon Mungunsukh, Kevin Chuang, Iren Horkayne-Szakaly, Francis G. O'Connor, Patricia A. Deuster

**Affiliations:** ^1^Consortium for Health and Military Performance, Department of Military and Emergency Medicine, Hébert School of Medicine, Uniformed Services University, 4301 Jones Bridge Rd., Bethesda, MD 20184, USA; ^2^Department of Anesthesiology, University of Minnesota, 420 Delaware St SE, Minneapolis, MN 55455, USA; ^3^Department of Anesthesiology, Hébert School of Medicine, Uniformed Services University, 4301 Jones Bridge Rd., Bethesda, MD 20814, USA; ^4^Joint Pathology Center, Defense Health Agency, 606 Stephen Sitter Ave, Silver Spring, MD 20910, USA

## Abstract

Individuals with Sickle Cell Trait (SCT), generally considered a benign carrier state of hemoglobin S (HbAS), are thought to be at risk for exertional rhabdomyolysis and hematuria, conditions that can also be caused by various other acquired and inherited factors. We report an SCT positive service member with an exertional rhabdomyolysis event, recurrent hematuria with transient proteinuria, and episodic burning pain in the lower extremities. Clinical and genetic studies revealed the multifactorial nature of his complex phenotype. The service member was taking prescription medications known to be associated with exertional rhabdomyolysis. He carried a pathogenic mutation,* NPHS2* p.V260E, reported in nephropathy and a new variant p.R838Q in* SCN11A*, a gene involved in familial episodic pain syndrome. Results suggest that drug-to-drug interactions coupled with the stress of exercise, coinheritance of HbAS and* NPHS2* p.V260E, and p. R838Q in* SCN11A* contributed to exertional rhabdomyolysis, recurrent hematuria with proteinuria, and episodic pain, respectively. This case underscores the importance of comprehensive clinical and genetic evaluations to identify underlying causes of health complications reported in SCT individuals.

## 1. Introduction

Sickle Cell Trait (SCT) is a carrier state (HbAS) of a hemoglobin mutation that causes Sickle Cell Disease, one of the most common monogenic inherited blood disorders in humans. It is estimated that about 300 million individuals worldwide have SCT [1]. In the United States, 7.3% of African Americans, 0.7% of Hispanics, and 0.3% Caucasians are carriers of HbAS [2].

Although SCT is generally considered a benign carrier state, it has been associated with a number of health complications such as exertional rhabdomyolysis and hematuria [1, 3, 4]. Exertional rhabdomyolysis is a metabolic event characterized by the release of muscle contents into the circulation due to exercise related skeletal muscle breakdown. Typically, exertional rhabdomyolysis manifests as muscle pain and weakness, with transient elevation (>5-10 x normal) in serum creatine kinase (CK), with or without myoglobinuria [5]. The development of exertional rhabdomyolysis depends on numerous risk factors that can be broadly divided into acquired and inherited. Acquired causes include injuries, medications, supplements, infections, and overexertion [5, 6]. Inherited etiologies include genetic abnormalities in the glycolytic and the fatty acid oxidation pathways, and in the mitochondrial respiratory chain [5].

Hematuria in SCT individuals is typically painless and presents as microscopic or gross bleeding. Hematuria thought to occur due to vasoocclusion events that can lead to microinfarctions and renal papillary necrosis [1, 3]. As with exertional rhabdomyolysis, hematuria can rise from metabolic and/or environmental factors such as dehydration, strenuous exercise, and acidosis. Once these precipitating factors are eliminated, there is usually no recurrence; however, if hematuria persists and its etiology is uncertain, other factors should be considered. A number of studies have demonstrated the effect of genetic variants in renal phenotypes in SCT individuals [7, 8].

Pain in SCT individuals has been reported in association with splenic infarction and pulmonary embolism [1, 3]. In these settings, SCT carriers experience acute pain in chest or left upper abdomen which is again thought secondary to HbAS driven vasoocclusive event. Complications associated with splenic infarction and pulmonary embolism are relatively rare and seen mostly under hypoxic conditions of high altitude [9, 10].

Episodic pain reflecting a neurological disorder is clinically distinct from pain associated with HbAS. A growing body of evidence demonstrates a monogenic origin, specifically, the involvement of channelopathies in pain [11]. Recently, autosomal dominant mutations in* SCN11A*, which encodes Nav1.9, a voltage-gated sodium channel subunit alpha type 9, have been found in pain syndromes, including episodic familial pain syndrome [11–13].

We report clinical and genetic studies of a service member with SCT who presented with exertional rhabdomyolysis, recurrent hematuria with transient proteinuria, and episodic burning pain in the lower extremities. Study results revealed the multifactorial nature of his complex clinical symptoms with involvement of both acquired and inherited causes. Patient was reported taking prescription medications known to be associated with exertional rhabdomyolysis. Whole exome sequencing (WES) identified a pathogenic mutation in* NPHS2* associated with nephropathy [14] and likely pathogenic variant in* SCN11A*. Our study results underscore the importance of detailed clinical and genetic evaluations to identify underlying causes of health complications reported in SCT individuals.

## 2. Case Description

A 33-year-old African American male service member with SCT presented with an episode of myalgia, muscle stiffness, and a peak CK of 18,867U/L after exercise. His clinical history was significant for chronic exercise associated hematuria, transient proteinuria with creatinine elevation, and severe episodic pain in his lower extremities. He stated that his symptoms had begun four years earlier when he developed shortness of breath and muscle pain and was unable to complete a two-mile run. He was evaluated and found to have hematuria. Since then, he has had multiple episodes of hematuria after exertion with severe muscle pain and stiffness lasting 3-4 days. He reported hydrating well during or following exercise but had also noticed that his calves had become smaller over the years, despite exercise. He had undergone an extensive work-up for the hematuria, to include cystogram and renal ultrasound, but without any definitive diagnosis. He denied a family history of muscle problems and/or adverse reactions to anesthesia; however, a family history of pain or hematuria is unknown. Underlying inflammatory myopathy was ruled out, but the service member was placed on restricted physical activity. However, even with his relative inactivity, he complained of burning pain in his calves and shoulder stiffness two to three times a week. In addition, his health record indicated history of hypertension and depression. Active prescription medications included amlodipine (10-40mg), simvastatin (20mg), sertraline (100mg), and indomethacin (25mg).

Muscle histology showed minimal nonspecific changes (Supplemental Material, Fig.  S1). Muscle enzymes were within reference ranges. Electromyography was negative for myopathy. Details regarding nerve conduction studies were not available from clinical history.

### 2.1. Genetic Study

WES, variants identification and analysis were performed as described previously [15]. Briefly, variants were filtered for minor allele frequency of <0.1-0.01% in the general population. Nonsynonymous, splice, stop gain, and stop loss variants were prioritized. A number of* in silico* algorithms including SWIFT, PolyPhen, and Mutation Tester were utilized to predict the effect of identified variants. The pathogenicity of the variants was assessed per American College of Medical Genetics (ACMG) guidelines [16]. Paralogue annotation method [17] was used to further assess pathogenic effect of variants. The structure of Nav1.9 was predicted and visualized as described by Omasits U. et al. [18]. Sanger sequencing was used to confirm variants identified by WES.

## 3. Results

**Figure 1:**
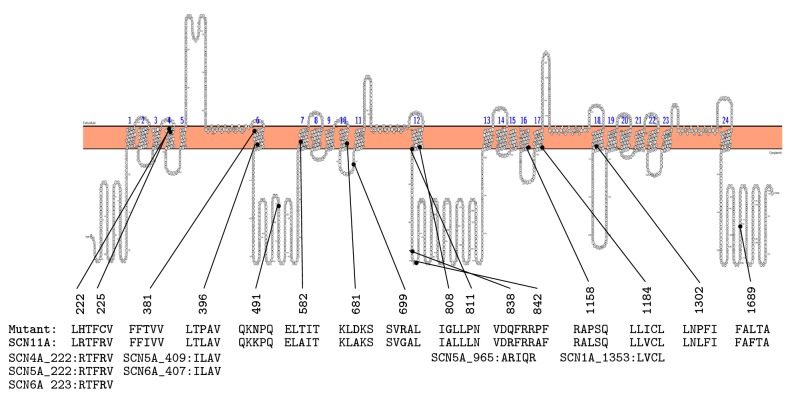
Locations and paralogue analysis of* SCN11A *variants associated with pain diseases in the Nav1.9 channel. The Nav1.9 channel, like other sodium channels, is composed of 24 transmembrane segments organized into four domains. Mutated residues are in bold, black dots represent locations of mutated residues in the channel. Amino acid residue number is given above mutated residue. Paralogue sodium channels along with mutated residues and their numbers are given below equivalent residues in* SCN11A.*

Prior to WES analysis, common pathogenic variants in* PYGM*,* CPT2*,* AMPD1,* and* RYR1*, genes frequently associated with ER, were analyzed with negative results. WES results for other genes associated with metabolic myopathies implicated in rhabdomyolysis were negative. WES identified seven variants that fulfilled stringent analysis criteria (Table 1). Of deleterious variants identified in this study, p. V260E variant in* NPHS2 *was previously reported as a pathogenic mutation (Fig.  S2) [14]. Remaining variants were analyzed in detail (Supplementary material). Based on information regarding encoded protein function, disease association, disease inheritance pattern, the degree of pathogenicity predicted by various* in silico* methods, and paralogue analysis, p. R838Q in* SCN11A *was selected as a candidate pathogenic variant contributing to patient's phenotype. The p. R838Q in* SCN11A *was predicted to be damaging by various analysis tools used in this study. Protein alignment and paralogue annotation analyses showed that arginine residue at 838 position of* SCN11A *is highly conserved among various species and also between different subunits of voltage-gated sodium channels (Fig.  S3). Importantly, paralogous mutations in another member of voltage-gated sodium channels, specifically* SCN5A*, have been associated with cardiomyopathies [19, 20]. These results suggest that p. R838Q in* SCN11A* is likely pathogenic variant. Paralogue analysis was extended to all known disease-associated variants in* SCN11A* as well as variant locations in Nav1.9 (Figure 1, Table S).

## 4. Discussion

Exertional rhabdomyolysis often occurs in military service members engaged in strenuous physical activity. It has many potential causes that can be divided into acquired and inherited [5, 21]. The service member in this study, who was SCT positive, presented as having had a single episode of exertional rhabdomyolysis with severe muscle pain, peak CK of 18,867U/L, and hematuria after exercise. Muscle enzymes and histopathology were essentially normal, thus ruling out metabolic and mitochondrial myopathies commonly associated with exertional rhabdomyolysis [5, 21]. This result was consistent with genetic results, which were negative for any predicted or known pathogenic variants associated with rhabdomyolysis. Detailed analysis of the clinical history revealed use of several prescription medications including amlodipine, simvastatin, and sertraline. Statin use is known to be associated with mild muscle complaints that in combination with amlodipine significantly increase the risk for acute rhabdomyolysis [6]. As a result, the daily dose of simvastatin is restricted to ≤20mg when taking amlodipine. Patient was taking simvastatin within the restricted dosage, but he was also taking sertraline (100mg), antidepressant selective serotonin reuptake inhibitor [22]. The daily dose of sertraline reported in association with exertional rhabdomyolysis ranges from 50-150 mg [22–24]. Rhabdomyolysis has also been precipitated by concomitant use of sertraline and other drugs, including amlodipine and simvastatin [22, 24]. In most reported cases, strenuous exercise appears to be a common thread in sertraline-induced rhabdomyolysis [22, 23]. These results suggest that sertraline and/or drug-to-drug interactions, together with the stress of exercise, may have contributed to the development of exertional rhabdomyolysis in this case.

The service member's history of recurrent hematuria and transient proteinuria triggered by exercise is significant. WES analysis revealed that the warfighter was a carrier of p. V260E, a known pathogenic variant in* NPHS2* encoding Podocin, a glomerular podocyte protein that serves a key role in the regulation of glomerular permeability [14, 25]. Podocin abnormality typically results in dysfunction of the glomerular filtration barrier and development of proteinuria. Biallelic mutations in* NPHS2 *cause a nephropathy known as steroid-resistant nephrotic syndrome (SRNS), which is characterized by massive proteinuria, hypoalbuminemia, and edema [14]. The inheritance of pathogenic* NPHS2 *p. V260E mutation likely contributed to the patient's recurrent hematuria and proteinuria. Given SCT status and history of recurrent exercise-induced hematuria and proteinuria, it is likely that coinheritance of p. V260E in* NPHS2* and HbAS likely have cumulative effects and compromised kidney function upon exertion.

Another key finding was the identification of the* SCN11A* p. R838Q variant. Patient had reported severe and burning pain in his calves triggered by exercise. His pain, localized predominantly in the distal lower extremity, was distinct from the abdominal and chest pain due to splenic infarction and pulmonary embolism in SCT individuals [1, 3]. In addition, his leg pain was unique from exertional leg pain in SCT individuals we have previously seen clinically in its atypical presentation in lasting days in duration. Pathogenic variants in* SCN11A *are associated with a range of autosomal dominant syndromic pain diseases such as episodic pain, painful neuropathy, and insensitivity to pain [12, 13, 26]. The p. R838Q variant located in close proximity to three other* SCN11A* pathogenic variants, including A808G and A842P, reported in association with episodic pain and painful neuropathy respectively [12, 13]. The p. R838Q variant like other pathogenic variants, p. R225C, p. I381T, p. L396P and p. V1184A, in* SCN11A* was annotated with paralogue mutations in functionally similar members of the voltage-gated sodium channel family (Figure 1). There is also striking resemblance between episodic pain described in* SCN11A* families and the pain phenotype manifested in the reported case.* SCN11A*-associated episodic pain is characterized by severe burning pain predominantly in the lower extremities [12, 13]. Pain occurs episodically, typically once every 2-5 days and can be triggered or exacerbated by fatigue or heavy exercise. We suggest that p. R838Q variant, similarly with other* SCN11A* pathogenic variants, may cause neuronal hyperexcitability and lead to the service member's episodes of severe burning calf pain. Further studies of the functional effect of this variant in episodic pain are warranted.

Development of exertional rhabdomyolysis and hematuria in SCT individuals is thought to occur due to polymerization of HbAS and red blood cell sickling [1, 3]. However, whether HbAS-related red blood sickling is the cause of these complications remains controversial. Exercise studies have demonstrated no difference in muscle microvasculature, blood rheology, and inflammatory factors between individuals with and without SCT [27]. Epidemiological studies have shown that exertional rhabdomyolysis and hematuria occur in 1.2% and 2.5% of African Americans with SCT respectively [28, 29]. The fact that not all SCT carriers develop exertional rhabdomyolysis and hematuria indicates a variable degree of risks and expressions of these complications among SCT individuals. Importantly, this highlights a role of other environmental and genetic factors as presented in the case under the study. The service member was taking a combination of drugs known to be associated with an increased risk for exertional rhabdomyolysis suggesting that drug-to-drug interactions, together with the stress of exercise, may have contributed to the development of exertional rhabdomyolysis in this case. Coinheritance of HbAS and* NPHS2* p.V260E, rather than sickling alone, may be the underlying cause of recurrent hematuria and transient proteinuria. The likely pathogenic variant p. R838Q in* SCN11A* may explain, in part, the patient's episodic burning pain affecting his calf muscles.

In conclusion, we identified a SCT positive case who presented with exertional rhabdomyolysis, recurrent hematuria, and episodic burning pain in the lower extremities. Clinical and genetic studies revealed the multifactorial nature of his clinical presentations with the role of both acquired and inherited factors in the observed phenotypes. This case underscores the importance of comprehensive clinical and genetic evaluations to identify underlying causes of health complications reported in SCT individuals.

## Figures and Tables

**Table 1:** Exome sequencing results. Findings are presented by the degree of pathogenicity predicted by various methods.

**Gene **	**Variant**	**Variant ID** ^a^	**ExAC freq. (**%**)**	**Variant impact prediction** ^b^		**ACMG class**	**Associated disease Inheritance Pattern** ^c^
				Sift PolyPhen	Mutation Tester		
*NPHS2 *	V260E	rs775006954	0.005	Del.	GScore: 121 Pp: 1.0	Pathogenic	Steroid Resistant Nephrotic Syndrome, AR

*TTN *	T587Hfr11Ter	NA	NR	LoF	GScore: NA Pp: 1.0	VUS	Various cardiac and muscle disorders, AD/AR

*SCN1A *	R604C	rs148371904	0.002	Del.	GScore: 180 Pp: 1.0	VUS	Epilepsy, Familial Migraine, AD

*SCN11A *	R838Q	rs149681198	0.007	Del.	GScore: 43 Pp: 0.97	Likely pathogenic	Familial Episodic Pain, AD

*SPTBN4 *	R527W	NA	NR	Del.	GScore: 101 Pp: 0.99	VUS	Congenital myopathy with neuropathy, AR

*HSPG2 *	R2196W	rs566319401	0.003	Del.	GScore: 101 Pp: 0.77	VUS	Schwartz-Jampel syndrome, AR

*DSG *	L171F	rs199926617	0.002	Del.	GScore: 22 Pp: 0.50	VUS	Arrhythmogenic Right Ventricular Dysplasia, AD/AR

^a^NA or NR – Not available or not reported

^b^Del. - Deleterious; LoF – Loss of function; GScore: Grantham Score scores substitutions according to the degree of the physico- chemical difference between the original and the new amino acid; Pp: The probability of the prediction, a value close to 1 indicates a high probability of pathogenicity.

^c^AD – Autosomal Dominant; AR- Autosomal Recessive.

## Supplementary Materials

Supplementary Materials Supplementary materials include muscle histopathology images (Fig. S1), integrated view of exome sequencing results (Fig. S2), amino acid alignments of regions with substituted residues in the voltage-gated sodium channels (Fig. S3), and genetic variant analysis and citations.
