# Supplementary material for: Multifactorial Origin of Exertional Rhabdomyolysis, Recurrent Hematuria, and Episodic Pain in a Service Member with Sickle Cell Trait
Source: Case Rep Genet. 2018 Nov 7;2018:6898546. doi: 10.1155/2018/6898546 (PMC6247656; doi:10.1155/2018/6898546)
Supplement: Supplementary Materials — include muscle histopathology images (Fig. S1), integrated view of exome sequencing results (Fig. S2), amino acid alignments of regions with substituted residues in the voltage-gated sodium channels (Fig. S3), and genetic variant analysis and citations. [file 6898546.f1.pdf]

Supplemental Material

Multifactorial Origin of Exertional Rhabdomyolysis, Recurrent Hematuria and Episodic Pain in a Service Member with Sickle Cell Trait

Sambuughin N., Ren M., Capacchione J.F., Mungunsukh O., Chuang K., Horkayne-Szakaly I., O'Connor F.G., Deuster P.A.

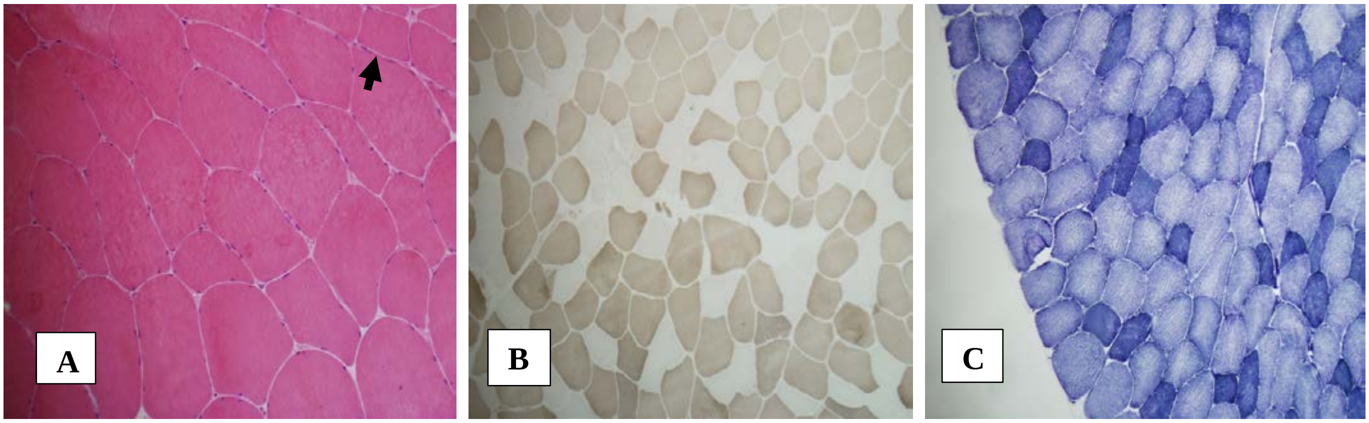

Figure S1. **A**. Haematoxylin and Eosin staining demonstrates mild variation in fiber size due to rare atrophic fibers (an arrow). **B.** ATP-ase staining shows normal distribution of fiber types. **C**. The nicotinamide adenine dinucleotide-tetrazolium reductase stain reveals minimal subsarcolemmal crescents.

Genetic variant analysis

We determined that *SPTBN4* p.R527W, *HSPG2* p. R2196W, and *DSG2* p.L171W variants were not contributory to the patient's phenotype, based on information regarding encoded protein function, disease association, disease inheritance pattern, and/or the degree of pathogenicity predicted by various *in silico* methods.

The loss of function (LoF) variant, p. T587Hfr11Ter in *TTN* was predicted to be pathogenic. Variants in *TTN* - including LoF variants - are associated with various cardiac and skeletal muscle disorders [1-3]. *TTN* encodes Titin, a giant sarcomeric protein; about 1-3% of individuals in the general population carry a LoF variant in *TTN* [1, 2]. The location of the variant appears to

be important for the determination of variant pathogenicity. Whereas variants in the A-band region are predominantly associated with cardiomyopathies, variants in the C-terminal region are preferentially mutated in skeletal muscle disorders [1-3]. The variant locates in the N-terminal region of *TTN* where numerous variants including LoF variants are found in the general population [2]. Based on these results, we determined that the impact of *TTN* p. T587Hfr11Ter variant on the patient's phenotype was highly unlikely.

The patient carried variants in two members of the voltage-gated sodium channels: p. R604C in *SCN1A*, encoding Nav1.1, and p. R838Q in *SCN11A,* encoding Nav1.9. Both variants replaced the arginine residues that are conserved among *SCN1A* and *SCN11A* between species (Fig. S2. A). However, these residues showed different degrees of evolutionary conservation when compared to other members of this protein family. The p. R604 residue in *SCN1A* was only partially conserved. Furthermore, there was no homology of this region containing p. R604C in *SCN1A* with two other members (Fig. S2. B). In contrast, p. R838 residue in *SCN11A* was completely conserved among all nine members of the protein family (Fig. S2. B). We then analyzed whether other sodium channels where variants replacing amino acid residues equivalent to *SCN1A* p. R604 and *SCN11A* p. R838 residues were pathogenic or associated with human diseases. Results were negative for *SCN1A* p. R604 residue, whereas three different variants in *SCN5A* changing the arginine at position 965 equivalent to p. R838 residue in *SCN11A* are known to be associated with various cardiac diseases. Disease associated *SCN11A* were reviewed [4-10] and analyzed by paralogue annotation method.

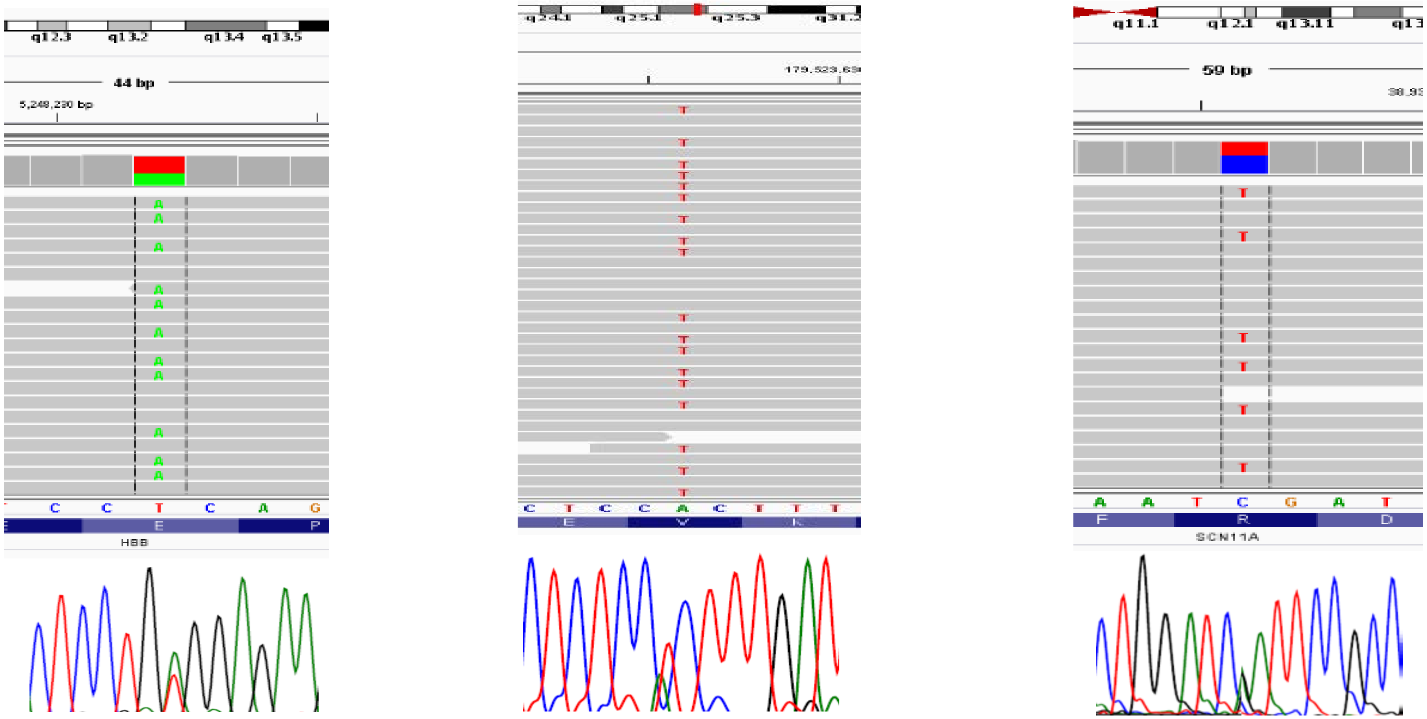

HBB: p. E7V

NPHS2: p. V260E

SCN11A: p. R838Q

Figure S2. Genetic results. Integrated view of exome sequencing reads presented at the top. Sequence alignments are shown as gray polygons. Single nucleotide variants mismatching the reverence sequences indicated by color in integrated view. Sanger sequencing was used to confirm variants. Sanger sequencing chromatograms and genetic variants are presented below aligned exome sequence reads.

A.

	SCN1A:	SCN11A:
	604	838
**Variant**	DNES**C**RDSL	LALD**Q**FRRA
**Human**	DNES**R**RDSL	LALD**R**FRRA
**Dog**	DNES**R**RDSL	LALD**R**FHRV
**Chicken**	DNDS**R**RDSL	LALD**R**FRRV
**Rat**	DNES**R**RDSL	LALD**R**FRRA
**Mice**	DNES**R**RRSL	LALD**R**FRRA

B.

Gene	Protein	604	838
**Variant**		DNES**C**RDSL	LALD**Q**FRRA
**SCN1A**	**Nav1.1**	DNES**R**RDSL	IAVD**R**MHKG
**SCN11A**	**Nav1.9**	---------	LALD**R**FRRA
**SCN2A**	**Nav1.2**	DNDS**R**RDSL	IAVG**R**MQKG
**SCN3A**	**Nav1.3**	DSES**R**RDSL	IALG**R**MQKG
**SCN4A**	**Nav1.4**	---------	IAIG**R**IKLG
**SCN5A**	**Nav1.5**	ESES**H**HTSL	LALA**R**IQRG
**SCN8A**	**Nav1.6**	ESEG**R**RDSL	ISVI**R**IKKG
**SCN9A**	**Nav1.7**	DNES**R**RGSL	IAVT**R**IKKG
**SCN10A**	**Nav1.8**	DHES**H**RGSL	VALA**R**IQVF

Figure S3. A. Amino acid alignment of *SCN1A* and *SCN11A* shows p. R604 and p. R838 residues are conserved between species. **B.** Paralogue annotation of the voltage gated sodium channel family members. The R604 residue and surrounding region in *SCN1A* has no homology with *SCN4A* and *SCN11A*.

Table S. Summary of *SCN11A* variants reported in pain syndrome and paralogue annotation of variants.

SCN11A variant	Associated pain syndrome	# reported families	Allele frequency (%) in ExAc	Paralogue gene	Paralogue variant	Paralogue Disease
R222H	Episodic pain	6 families	Not Available	-	-	
R222S	Episodic pain	1 family	Not Available	-	-	
R225C	Episodic pain	2 families	0.001	SCN4A	R222W	Hypokalemic Periodic Paralysis
				SCN5A	R222Q	Long QT syndrome, Brugada syndrome
				SCN6A	R223G	Epileptic encephalopathy
I381T	Painful neuropathy	2 cases	Not Available	-	-	
L396P	Insensitivity to pain	1 case	Not Available	SCN5A	L409V	Long QT syndrome
				SCN6A	L407F	Epileptic encephalopathy
K491N	Painful neuropathy	2 cases	Not Available	-	-	
A582T	Painful neuropathy	2 cases	0.01	-	-	
A681D	Painful neuropathy	1 case	Not Available	-	-	
G699R	Painful neuropathy	1 case	0.02	-	-	
A808G	Episodic pain	1 family	Not Available	-	-	
L811P	Insensitivity to pain	2 cases	Not Available	-	-	
R838Q	Episodic pain	1 case, this study	0.007	SCN5A	R965C	Brugada syndrome
				SCN5A	R965H	Brugada syndrome
				SCN5A	R965L	Long QT syndrome
A842P	Painful neuropathy	1 case	Not Available	-	-	
L1158P	Painful neuropathy	2 cases	0.05	-	-	
V1184A	Cold-aggravated episodic pain	1 family	Not Available	SCN1A	V1353L	Generalized epilepsy with febrile seizures
L1302P	Insensitivity to pain	1 family	Not Available	-	-	
F1689L	Painful neuropathy	1 case	0.02	-	-	

References

1. Herman, D.S., et al., *Truncations of titin causing dilated cardiomyopathy.* N Engl J Med, 2012. **366**(7): p. 619-28.
2. Tabish, A.M., et al., *Genetic epidemiology of titin-truncating variants in the etiology of dilated cardiomyopathy.* Biophys Rev, 2017. **9**(3): p. 207-223.
3. Savarese, M., et al., *Interpreting Genetic Variants in Titin in Patients With Muscle Disorders.* JAMA Neurol, 2018.
4. Dib-Hajj, S.D., J.A. Black, and S.G. Waxman, *NaV1.9: a sodium channel linked to human pain.* Nature Reviews Neuroscience, 2015. **16**: p. 511.
5. Okuda, H., et al., *Infantile Pain Episodes Associated with Novel Nav1.9 Mutations in Familial Episodic Pain Syndrome in Japanese Families.* PLoS One, 2016. **11**(5): p. e0154827.
6. Han, C., et al., *Familial gain-of-function Nav1.9 mutation in a painful channelopathy.* J Neurol Neurosurg Psychiatry, 2017. **88**(3): p. 233-240.
7. King, M.K., et al., *Pain insensitivity: distal S6-segment mutations in NaV1.9 emerge as critical hotspot.* Neurogenetics, 2017. **18**(3): p. 179-181.
8. Leipold, E., et al., *Cold-aggravated pain in humans caused by a hyperactive NaV1.9 channel mutant.* Nat Commun, 2015. **6**: p. 10049.
9. Huang, J., et al., *Sodium channel NaV1.9 mutations associated with insensitivity to pain dampen neuronal excitability.* J Clin Invest, 2017. **127**(7): p. 2805-2814.
10. Zhang, X.Y., et al., *Gain-of-function mutations in SCN11A cause familial episodic pain.* Am J Hum Genet, 2013. **93**(5): p. 957-66.
